# Development and validation of a machine learning-based diagnostic model for obstructive coronary artery disease in hypertensive patients using composite inflammatory and lipid markers

**DOI:** 10.3389/fcvm.2026.1922999

**Published:** 2026-09-22

**Authors:** Jiajing Liu, Jinzhu Yin, Xiaoli Wang, Jiayuan Song, Yunfan Liu, Liping Chang

**Affiliations:** 1 Department of Cardiology, Jilin Provincial Hospital of Traditional Chinese Medicine, Changchun, Jilin, China; 2 Department of Geriatric Medicine, Jilin Provincial Hospital of Traditional Chinese Medicine, Changchun, Jilin, China

**Keywords:** hypertension, inflammatory index, lipid ratio, machine learning, obstructive coronary artery disease, risk stratification

## Abstract

**Background:**

Hypertensive patients face high risk of obstructive coronary artery disease (CAD), but early diagnosis is limited by costly or invasive gold-standard imaging. Composite inflammatory and lipid markers from routine blood tests may offer a low-cost alternative. We aimed to develop and validate a machine learning model using such markers for obstructive CAD in hypertensive patients.

**Methods:**

This retrospective cohort comprised 427 hypertensive patients treated at the Affiliated Hospital of Changchun University of Chinese Medicine between 2023 and 2025. Of these individuals, 195 patients (45.7%) received a diagnosis of obstructive coronary artery disease, characterized by coronary artery diameter stenosis equal to or greater than 50% as confirmed by coronary angiography. Through the integration of Boruta feature selection and variance inflation factor analysis, a set of four key diagnostic variables was identified: age, dyslipidemia, Lymphocyte-to-monocyte ratio (LMR), and Monocyte-to-HDL-C ratio (MHR). The dataset was partitioned into training and validation subsets at a ratio of 7:3. Five distinct machine learning models were constructed, and model performance was assessed using metrics such as the area under the curve (AUC), accuracy, F1 score, calibration, and SHAP analysis for both evaluation and interpretation of outcomes.

**Results:**

The support vector machine (SVM) model demonstrated the highest generalization performance, achieving a validation AUC of 0.786 (95% CI: 0.701, 0.862), an accuracy of 0.756, and an F1 score of 0.73. LMR exhibited a protective effect with a linear correlation, whereas MHR was identified as a risk factor. Notably, the age of 60 years was a critical threshold for disease susceptibility.

**Conclusion:**

Developed in this study, the parsimonious support vector machine model incorporates age, dyslipidemia,LMR and MHR. It enables effective diagnostic identification of obstructive coronary artery disease among hypertensive patients via routine clinical indicators. Being interpretable and non-invasive, this model is suitable for first-line screening before expensive invasive imaging tests and helps implement early risk stratification in primary healthcare settings.

## 1 Introduction

Hypertension is one of the most common cardiovascular risk factors globally. Patients with hypertension face a 2–3 fold increased risk of developing coronary heart disease compared to their normotensive counterparts. Additionally, hypertension predisposes individuals to coronary atherosclerosis, which manifests as non-obstructive coronary artery disease, obstructive coronary artery disease, and diffuse vascular lesions at a high frequency (1). In hypertensive patients, coronary lesions are often diffuse and heavily calcified, significantly elevating the risks of myocardial infarction, heart failure, and cardiovascular mortality (2, 3).

The onset of obstructive coronary artery disease in these patients is typically insidious and presents with atypical symptoms, which are frequently misattributed to concurrent conditions such as blood pressure lability or left ventricular hypertrophy. This misattribution often results in a delayed diagnosis and subsequent adverse cardiovascular events (4). Accordingly, early and accurate identification of obstructive CAD among hypertensive populations has profound clinical and public health significance.

In recent years, readily accessible composite inflammatory indices (LMR, SIRI, AISI, etc.) and novel lipid ratios (NHHR, RC, etc.) have attracted extensive research attention, as they are closely linked to the inflammatory-lipid interactive mechanisms underlying atherosclerosis (5–7).

Existing models either depend on costly imaging-derived scores unsuitable for primary care or require >15 non-routine biomarkers (8). Current research does not adequately address the critical need for an accurate and interpretable diagnostic model that relies solely on easily obtainable composite inflammatory and lipid biomarkers, without the use of advanced imaging techniques. This gap presents a substantial barrier to early risk stratification and outpatient screening workflows for hypertensive patients. Consequently, there is an urgent need for a diagnostic tool that fully utilizes routine clinical data, effectively models non-linear relationships, and balances diagnostic accuracy with interpretability.

As a subfield of artificial intelligence, machine learning is particularly adept at analyzing high-dimensional multimodal clinical data. It can automatically identify complex non-linear interactions among clinical features, effectively reduce overfitting, and sustain stable diagnostic performance across diverse populations (9). Consequently, machine learning has become a fundamental methodology for developing diagnostic models in the field of precision medicine (10, 11). Machine learning is well-suited to address this gap because it can model non-linear interactions among a small set of routine biomarkers while maintaining interpretability through SHAP analysis, thereby balancing accuracy with clinical usability.

This study aimed to create and validate an interpretable machine learning model utilizing routinely collected clinical variables and atherosclerosis-related biomarkers to predict the presence of obstructive CAD in hypertensive patients. Hypertensive individuals were recruited for this retrospective cohort study, and composite inflammatory and lipid biomarkers were included. This study successfully determined the most suitable machine learning algorithm for forecasting the presence of developing obstructive coronary artery disease in this specific group.

## 2 Materials and methods

### 2.1 Research design and ethical considerations

This study protocol was approved by the Ethics Committee of the Affiliated Hospital of Changchun University of Chinese Medicine (Approval No. CCZYFYLL-SQ-2025shenzi-102) and carried out in accordance with the 2013 (seventh revision) revised Declaration of Helsinki. Written informed consent from patients was waived given the retrospective study design. All personal identifiable information related to patients was de-identified during data analysis.

### 2.2 Study population and eligibility criteria

This retrospective cohort study was conducted at a tertiary hospital in China. A total of 427 hypertensive patients were enrolled in the initial cohort, all of whom received treatment at the First Affiliated Hospital of Changchun University of Chinese Medicine between January 1, 2023 and June 30, 2025.

### 2.3 Eligibility criteria

#### 2.3.1 Inclusion criteria

1. Hypertension was defined as systolic blood pressure ≥ 140 mmHg and/or diastolic blood pressure ≥ 90 mmHg in accordance with the 2017 ACC/AHA Guidelines, confirmed by medical records or in-hospital documentation (12).
2. All patients underwent coronary computed tomographic angiography (CCTA), invasive coronary angiography (ICA), or other clinically indicated diagnostic examinations for the first time at our hospital to evaluate the presence of obstructive coronary artery disease (CAD).
3. Complete demographic data (age, gender), clinical history (smoking, alcohol consumption, duration of hypertension, diabetes mellitus, dyslipidemia), medication records (Antidiabetic drugs), and laboratory parameters required for calculating composite inflammatory and lipid indices (including LMR, NHHR, NLR, SIRI, AISI, etc.) were available. All laboratory indicators were measured using fasting venous blood samples collected on the second day of admission. Duration of hypertension was extracted from the first documented diagnosis in electronic medical records rather than patient self-report, minimizing recall bias. Although CCTA and invasive coronary angiography (ICA) differ in spatial resolution and invasiveness, both were used only to confirm the anatomical presence of ≥50% diameter stenosis. Prior studies demonstrate high concordance (*κ* > 0.80) between CCTA and ICA for obstructive CAD at this threshold, supporting their combined use for outcome ascertainment in this retrospective setting.
4. Age ≥ 18 years old.

#### 2.3.2 Exclusion criteria

1. Patients with a prior diagnosis of coronary artery disease (CAD) confirmed by CCTA, invasive coronary angiography, exercise stress testing, or typical angina symptoms, who had received corresponding treatments previously.

2. Patients with missing scan identifiers, unknown date of birth, or unavailable CCTA/ICA imaging data.

3. Patients complicated with severe hepatic or renal insufficiency, or malignant tumors.

4. Patients with CCTA images of insufficient quality for interpretative assessment.

5. Patients without available fasting venous blood laboratory results obtained on the second day of admission.

### 2.4 Outcome definition and ascertainment

Obstructive coronary artery disease (obstructive CAD) was defined according to the 2019 and 2024 European Society of Cardiology (ESC) guidelines on chronic coronary syndromes. The presence of a diameter stenosis of ≥50% in any major epicardial coronary artery (left main, left anterior descending, left circumflex, or right coronary artery) or one of its major branches on coronary angiography was considered to indicate obstructive CAD (13, 14).

### 2.5 Data collection and preprocessing

During data collection, this study complied with relevant medical research standards and protocols to ensure that the acquired data could adequately reflect patients' disease status and treatment profiles (15). The prespecified candidate diagnostic predictors were selected based on clinical plausibility, existing literature evidence regarding the risk of severe coronary stenosis, and the availability of clinical resources in participating hospitals (16).

Data were mainly extracted from electronic medical records, physician orders and laboratory testing systems. Collected information covered baseline demographics, disease-related clinical data and laboratory biomarkers, as detailed below: (1) Demographic characteristics: gender, age (years); (2) Medical history: history of heavy alcohol consumption, smoking history, duration of hypertension, diabetes mellitus, dyslipidemia; (3) Treatment profiles: use of antidiabetic agents; (4) Composite inflammatory and lipid indices:Neutrophil-to-Lymphocyte Ratio(NLR), Lymphocyte-to-Monocyte Ratio(LMR), Platelet-to-Lymphocyte Ratio(PLR), Systemic Inflammatory Response Index(SIRI), Castelli Risk Index II(CRI-II), Remnant Cholesterol(RC), Remnant Cholesterol to HDL-C Ratio(RC/HDL-C), Non-HDL-C to HDL-C Ratio(NHHR), Red Cell Distribution Width to Albumin Ratio(RAR), Neutrophil-to-Albumin Ratio(NPAR), Hemoglobin, Albumin, Lymphocyte, Platelet Score(HALP), Aggregate Index of Systemic Inflammation(AISI), Platelet-to-Albumin Ratio(PAR), Monocyte-to-Lymphocyte Ratio(MLR), Neutrophil-Lymphocyte to Monocyte Ratio(NLMR), Platelet-to-HDL-C Ratio(PHR), Lymphocyte-to-HDL-C Ratio(LHR), Monocyte-to-HDL-C Ratio(MHR).

SIRI=(absoluteneutrophilcount×absolutemonocytecount)/absolutelymphocytecount

HALP=(hemoglobin×albumin×absolutelymphocytecount)/plateletcount

AISI=(absoluteneutrophilcount×plateletcount×absolutemonocytecount)/absolutelymphocytecount

NLMR=(absoluteneutrophilcount×absolutelymphocytecount)/absolutemonocytecount

NHHR=(TC−HDL-C)/HDL-C

RC=TC−(LDL-C+HDL-C)

Eligible patients were randomly divided into a training cohort (300 subjects) and a validation cohort (127 subjects) at a ratio of 7:3. Stratified random sampling stratified by the presence or absence of obstructive coronary artery disease was adopted during allocation to balance the distribution of outcome events between the two groups. The missing data proportion of each variable was calculated as the total percentage of missing values across all observations. Predictors with a missing rate exceeding 30% were excluded from analysis. Multiple imputation by chained equations (MICE) was applied to continuous variables. Categorical variables had no missing values; had they occurred, we would have used mode imputation within the MICE framework (17). No variables exceeded the 30% missingness threshold; therefore, zero predictors were excluded on this basis. Data quality assurance included automated range checks, outlier flagging (values  >3 SD from the mean), and manual cross-verification of 10% of randomly selected records against source electronic medical records. Multiple imputation implemented in R software was applied to continuous variables to guarantee the integrity and accuracy of the dataset (18). In accordance with the 10 events per variable (EPV) rule, the minimum required number of positive cases in the training set was estimated based on the count of included diagnostic predictors (19). With 4 final predictors and 195 positive events, the training set (70% split, *n* ≈ 300) contained approximately 137 positive cases, yielding an EPV of 34.3 (137/4), well above the minimum threshold of 10.For baseline characteristic comparisons, the Benjamini-Hochberg false discovery rate (FDR) was applied across 27 candidate predictors. All FDR-adjusted *P* *<* *0.05* were considered significant. DeLong tests for AUC comparisons used a Bonferroni-adjusted *α* = 0.005 (0.05/10 pairwise comparisons).

### 2.6 Feature selection and model development

First, the Boruta algorithm was applied to conduct feature screening for all variables in the training set. Boruta is a random forest-based feature selection approach. It generates shadow features and compares their importance scores with original features to identify variables that are significantly associated with the study outcome. Boruta feature selection was performed using 5-fold cross-validation within the training set only. Shadow features were generated independently in each fold, and variables confirmed in ≥4 folds were retained (20).. Considering the potential multicollinearity among the screened composite indices, variance inflation factor (VIF) analysis was further performed on the selected variables, and only the most representative predictors were retained in the final model (21). Five machine learning algorithms were adopted in this study: logistic regression, support vector machine (SVM), artificial neural network (ANN), XGBoost, and decision tree. All models were trained on the training dataset, followed by performance evaluation on the validation set. The evaluation metrics consisted of discriminative performance (AUC with 95% confidence interval), decision curve analysis for clinical utility, calibration assessment, and comprehensive classification indicators including accuracy, precision, sensitivity, F1-score, specificity, Kappa coefficient, Youden's J statistic, positive diagnostic value (PPV), and negative diagnostic value (NPV). The optimal model was selected based on the comprehensive evaluation results. Comparisons of baseline characteristics between the NOCAD and OCAD groups were performed using the chi-square or Fisher's exact test for categorical variables and the independent t-test or Mann–Whitney *U* test for continuous variables. Given the simultaneous testing of multiple candidate predictors, false discovery rate (FDR) correction was applied using the Benjamini-Hochberg method to control for multiple comparisons. Statistical significance was defined as an FDR-adjusted *p* *<* *0.05*.

### 2.7 SHAP interpretability analysis

In addition, SHapley Additive exPlanations (SHAP) analysis was adopted to improve the interpretability of the model. First, the SHAP global feature importance bar plot was utilized to visualize the overall contribution of each feature to diagnostic outcomes. Second, the SHAP summary beeswarm plot was applied to simultaneously illustrate the direction and magnitude of each feature's effect on model prediction, as well as the corresponding feature values across all samples. Third, SHAP dependence plots were generated to explore the relationship between each feature and diagnostic risk. Finally, SHAP waterfall plots provided an intuitive illustration of how key features influence individual-level prediction results (22, 23). R software (version 4.4.3) and Python (version 3.12.7) were used for data analysis, model construction and validation. Statistical significance was defined as a two-tailed *P* *<* *0.05*.

### 2.8 Statistical analysis

Statistical analyses were performed using R software (version 4.4.3) and Python (version 3.12.7). Continuous variables conforming to a normal distribution were compared via the t-test or analysis of variance (ANOVA), and the results were expressed as mean ± standard deviation. Non-normally distributed data were analyzed using nonparametric tests, including the Wilcoxon rank-sum test and Kruskal–Wallis test, with outcomes presented as median and interquartile range (IQR). Categorical variables were compared using the chi-square test or Fisher's exact test and summarized as counts and percentages. A two-tailed *P* value < 0.05 was considered statistically significant (24, 25).

## 3 Result

### 3.1 Baseline characteristics

A total of 427 hypertensive patients were finally enrolled according to the inclusion and exclusion criteria, including 232 patients (54.33%) in the non-obstructive coronary artery disease (NOCAD) group and 195 patients (45.67%) in the obstructive coronary artery disease (OCAD) group. Among the 232 patients classified as having non-obstructive coronary artery disease (NOCAD), 158 underwent ICA and 74 were evaluated by CCTA only. In the OCAD group, all 195 patients underwent ICA (as CCTA-detected ≥ 50% stenosis was subsequently confirmed or managed via ICA in clinical practice). Compared with the NOCAD group, patients in the OCAD group were significantly older (median: 65.00 vs. 58.00, *P* *<* *0.001*) and had a longer duration of hypertension (median: 7.00 vs. 4.00, *P* *<* *0.001*), as well as a higher smoking rate (17.44% vs. 8.19%, *P* *=* *0.005*). Regarding comorbidities, the proportions of diabetes mellitus (35.38% vs. 22.41%, *P* *=* *0.004*) and dyslipidemia (93.85% vs. 64.22%, *P* *<* *0.001*) were markedly higher in the OCAD group. Meanwhile, the rates of antidiabetic drug use (29.23% vs. 16.38%, *P* *=* *0.002*) were also significantly elevated.

In terms of composite inflammatory and lipid indices, the levels of NLR, SIRI, MLR, NLMR, MHR, NPAR and AISI were significantly higher in the OCAD group than in the NOCAD group (all *P* *<* *0.05*). In contrast, LMR, PAR and HALP were obviously decreased in the OCAD group (all *P* *<* *0.05*). No significant differences were observed between the two groups in terms of gender, alcohol consumption, PLR, SII, LDL-C/HDL-C, RC, RC/HDL-C, NHHR, RAR, PHR and LHR (all *P* *>* *0.05*). (Table 1).

**Table 1:** Summarizes the intergroup comparison of baseline characteristics across all enrolled patients.

Characteristics	Overall	NOCAD	OCAD	*P*
	(*n* = 427)	(*n* = 232)	(*n* = 195)	
Gender (%)				
Male	213 (49.88)	108 (46.55)	105 (53.85)	0.146
Female	214 (50.12)	124 (53.45)	90 (46.15)	
Smoke (%)	53 (12.41)	19 ( 8.19)	34 (17.44)	0.005
Drunk (%)	50 (11.71)	27 (11.64)	23 (11.79)	1
Diabetes (%)	121 (28.34)	52 (22.41)	69 (35.38)	0.004
Dyslipidemia (%)	332 (77.75)	149 (64.22)	183 (93.85)	<0.001
Antihyperglycemic drugs (%)	95 (22.25)	38 (16.38)	57 (29.23)	0.002
Age (median [IQR])	62.00 [54.00, 69.00]	58.00 [49.00, 65.00]	65.00 [58.00, 70.00]	<0.001
Hypertension time (median [IQR])	5.00 [1.00, 10.00]	4.00 [1.00, 10.00]	7.00 [2.00, 10.00]	<0.001
NLR (median [IQR])	2.09 [1.55, 2.77]	2.02 [1.52, 2.56]	2.29 [1.61, 3.09]	0.001
LMR (median [IQR])	4.66 [3.50, 5.97]	5.00 [3.75, 6.36]	4.33 [3.36, 5.59]	0.001
PLR (median [IQR])	121.00 [98.05, 154.61]	120.65 [99.06, 150.00]	121.34 [96.09, 156.68]	0.693
SIRI (median [IQR])	0.82 [0.56, 1.16]	0.77 [0.52, 1.09]	0.92 [0.61, 1.34]	0.006
SII (median [IQR])	479.97 [338.90, 655.76]	473.90 [326.33, 608.28]	488.68 [347.77, 750.88]	0.083
CRI II (median [IQR])	2.27 [1.75, 2.86]	2.26 [1.82, 2.87]	2.29 [1.73, 2.77]	0.908
RC (median [IQR])	0.84 [0.59, 1.22]	0.87 [0.61, 1.27]	0.80 [0.54, 1.14]	0.171
RC/HDL-C (median [IQR])	0.31 [0.21, 0.43]	0.30 [0.21, 0.43]	0.31 [0.22, 0.42]	0.649
NHHR (median [IQR])	3.10 [2.34, 3.94]	3.26 [2.37, 3.95]	2.93 [2.32, 3.87]	0.447
RAR (median [IQR])	0.28 [0.27, 0.31]	0.28 [0.26, 0.30]	0.29 [0.27, 0.31]	0.066
NPAR (median [IQR])	0.09 [0.07, 0.11]	0.09 [0.07, 0.11]	0.10 [0.07, 0.12]	0.006
HALP (median [IQR])	50.23 [39.27, 64.89]	50.93 [40.73, 65.14]	49.24 [38.87, 63.63]	0.329
AISI (median [IQR])	190.68 [123.89, 290.67]	183.37 [110.52, 277.49]	208.13 [131.87, 321.02]	0.017
PAR (median [IQR])	5.21 [4.46, 6.02]	5.30 [4.66, 6.22]	5.11 [4.33, 5.87]	0.046
MLR (median [IQR])	0.21 [0.17, 0.27]	0.20 [0.15, 0.25]	0.23 [0.18, 0.29]	<0.001
NLMR (median [IQR])	2.38 [1.84, 3.05]	2.26 [1.78, 2.83]	2.58 [1.94, 3.37]	<0.001
PHR (median [IQR])	186.40 [154.95, 236.36]	186.09 [153.53, 237.04]	186.76 [155.05, 236.17]	0.928
LHR (median [IQR])	1.50 [1.13, 1.99]	1.50 [1.16, 1.98]	1.50 [1.10, 2.03]	0.687
MHR (median [IQR])	0.32 [0.24, 0.44]	0.31 [0.21, 0.43]	0.34 [0.26, 0.45]	0.019

*p*-values are false discovery rate (FDR) adjusted using the Benjamini-Hochberg method.

### 3.2 Feature selection

The Boruta algorithm was used for initial feature selection, and a total of 8 variables significantly associated with the outcome were identified: Dyslipidemia, Age, NLR, LMR, SIRI, MLR, NLMR and MHR (Figures 1, 2). Variance inflation factor (VIF) analysis was subsequently performed on these 8 variables to eliminate estimation bias caused by multicollinearity. A VIF value greater than 5 was defined as severe multicollinearity. As presented in Table 2, SIRI (VIF = 6.80), MLR(VIF = 23.39), NLR (VIF = 2,530.19) and NLMR (VIF = 2,781.38) exhibited extremely high multicollinearity with other variables and were therefore excluded.

**Figure 1:**
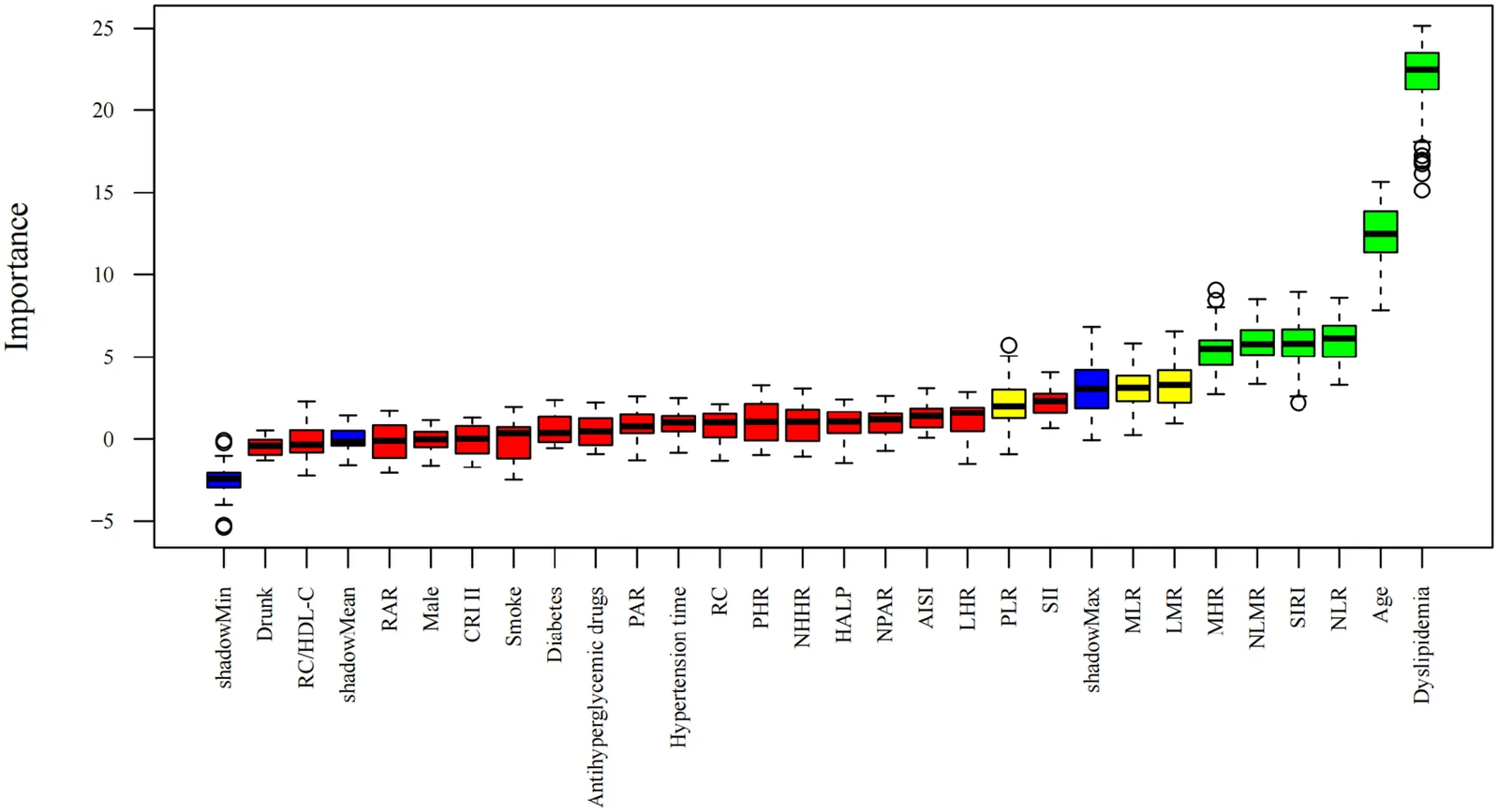
Box plot of feature importance for all candidate predictors.

**Figure 2:**
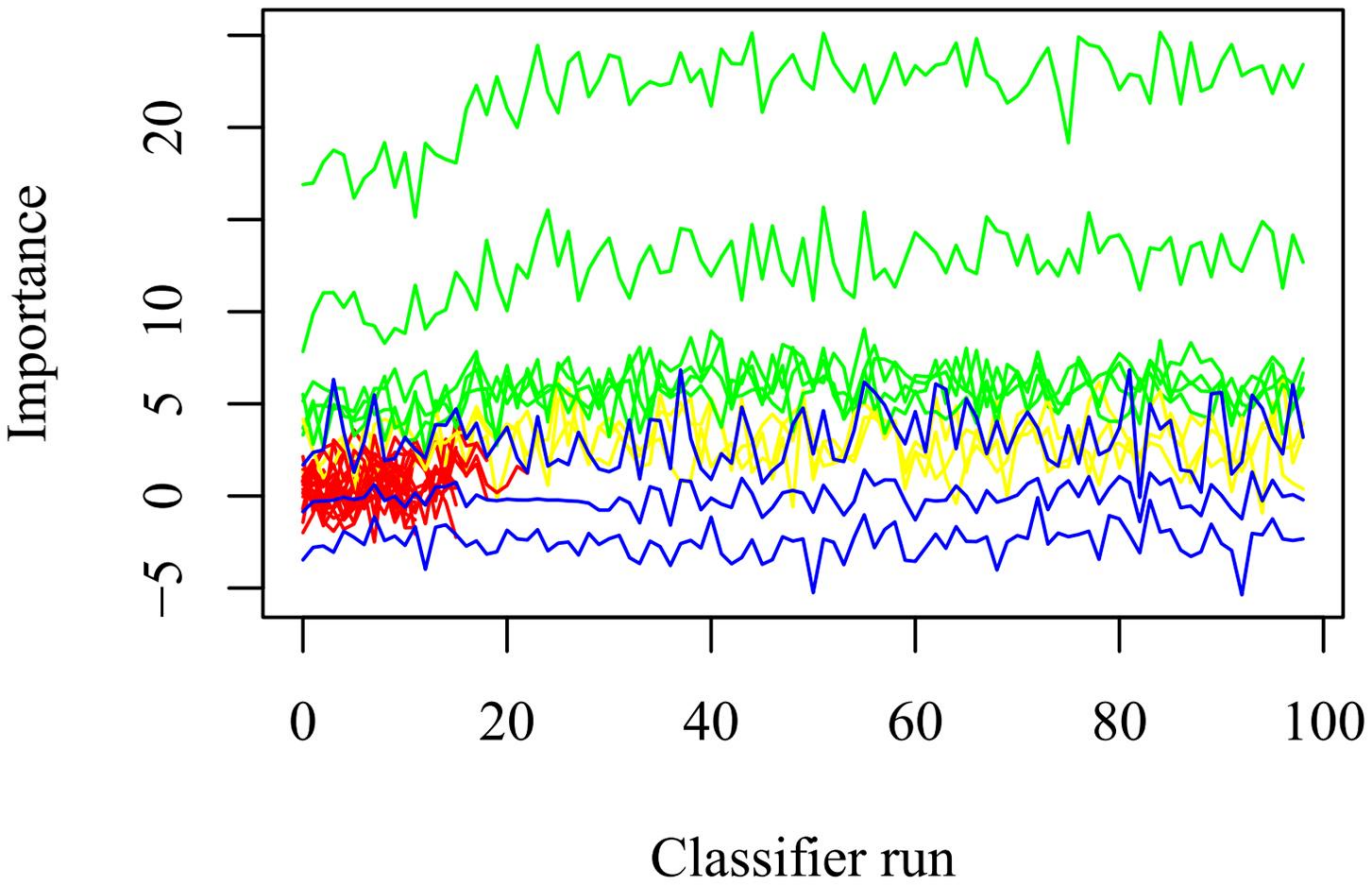
Dynamic feature importance trajectories across 100 independent classifier runs.

**Table 2:** Variance inflation factor (VIF) test.

Variable	Dyslipidemia	Age	MHR	LMR	SIRI	MLR	NLR	NLMR
Vif result	1.03	1.13	2.10	2.52	6.80	23.39	2,530.19	2,781.38

The VIF values of LMR (VIF = 2.52), MHR (VIF = 2.10), Age (VIF = 1.13), and Dyslipidemia (VIF = 1.03) were all below 5, indicating no obvious multicollinearity. These 4 indicators were ultimately selected as the core diagnostic variables. They cover distinct clinical dimensions including history of dyslipidemia, age, lipid-lowering treatment, immune-inflammatory status and lipoprotein ratio, with satisfactory independence and clinical interpretability.

### 3.3 Model development and validation

Based on the 4 features above, five machine learning models were established to predict the risk of obstructive coronary artery disease, including SVM, ANN, XGBoost and decision tree.

Hyperparameter tuning: In this study, the strategy of grid search combined with 5-fold cross-validation was adopted to perform hyperparameter tuning on four machine learning models: decision tree, XGBoost, ANN, and SVM. The optimization objective was the AUC value of the validation set, while taking into account the F1 score and calibration degree. The optimal combinations of hyperparameters for each model are as follows: Decision Tree: ccp_alpha = 0.01, max_depth = 5, max_features = “sqrt”, min_samples_split = 20; XGBoost: learning_rate = 0.01, max_depth = 3, n_estimators = 100, subsample = 0.6; Artificial Neural Network (ANN): activation = “logistic”, hidden_layer_sizes = (100, 100); Support Vector Machine (SVM): C = 1, kernel = “rbf”, gamma = 0.01, class_weight = None. Logistic regression employs the logit function of the statsmodels library to solve for parameters using maximum likelihood estimation (MLE), without imposing regularization penalties, and thus does not involve hyperparameter tuning. All tuning processes were completed through cross-validation within the training set without touching the validation set data to prevent data leakage.

The AUC values with 95% confidence intervals of each model in the training and validation sets are shown in Figures 3a, 3b. Decision curve analysis and calibration assessment of all models are presented in Figures 4a, 4b, 5a, 5b. To comprehensively evaluate model performance, we further calculated accuracy, precision, sensitivity, F1-score, specificity, Kappa coefficient, Youden's J statistic, positive diagnostic value (PPV) and negative diagnostic value (NPV). See Tables 3, 4.

**Figure 3:**
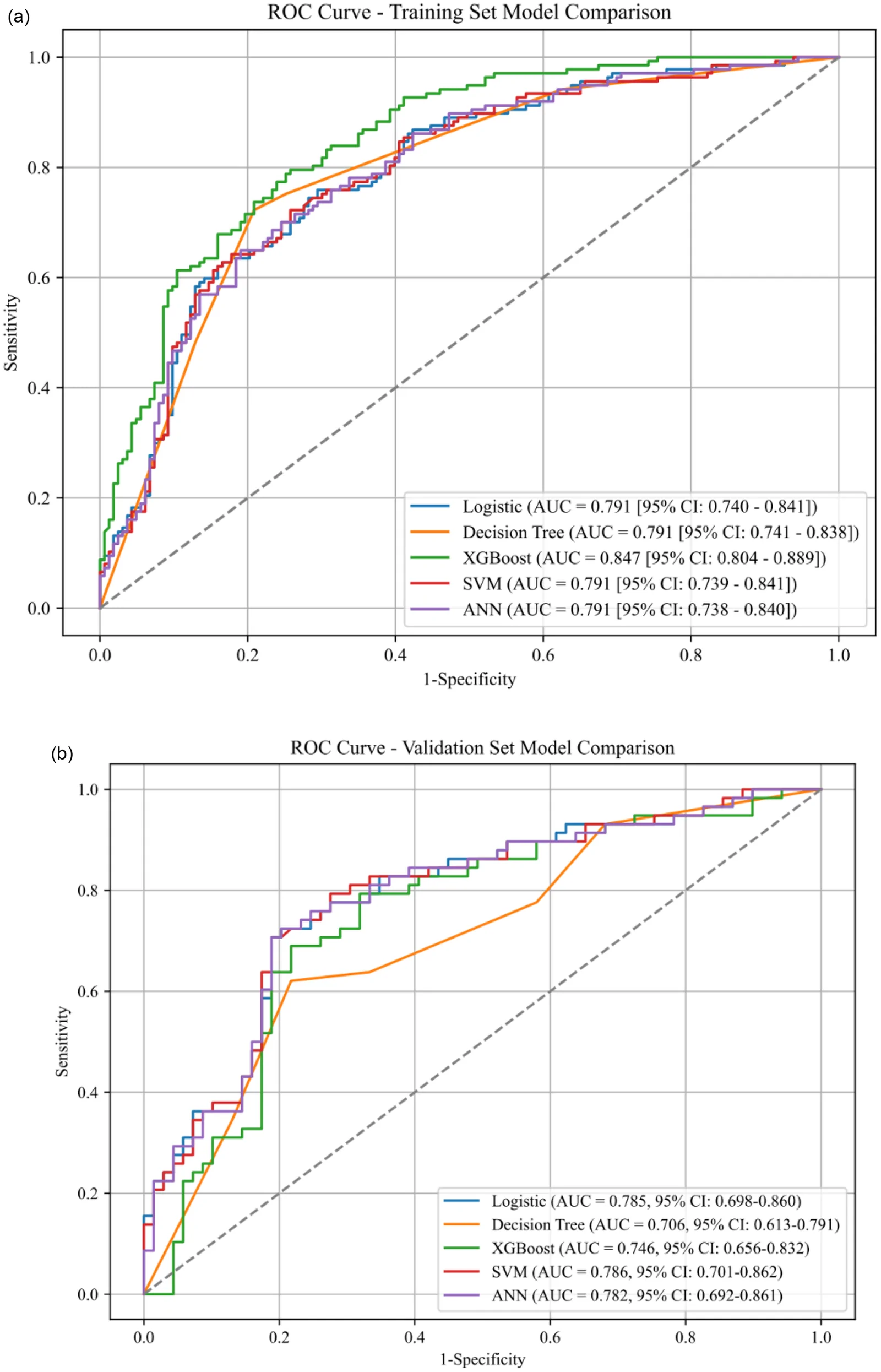
**(a)** ROC curves of five diagnostic models in the training set. **(b)** ROC curves of five diagnostic models in the validation set.

**Figure 4:**
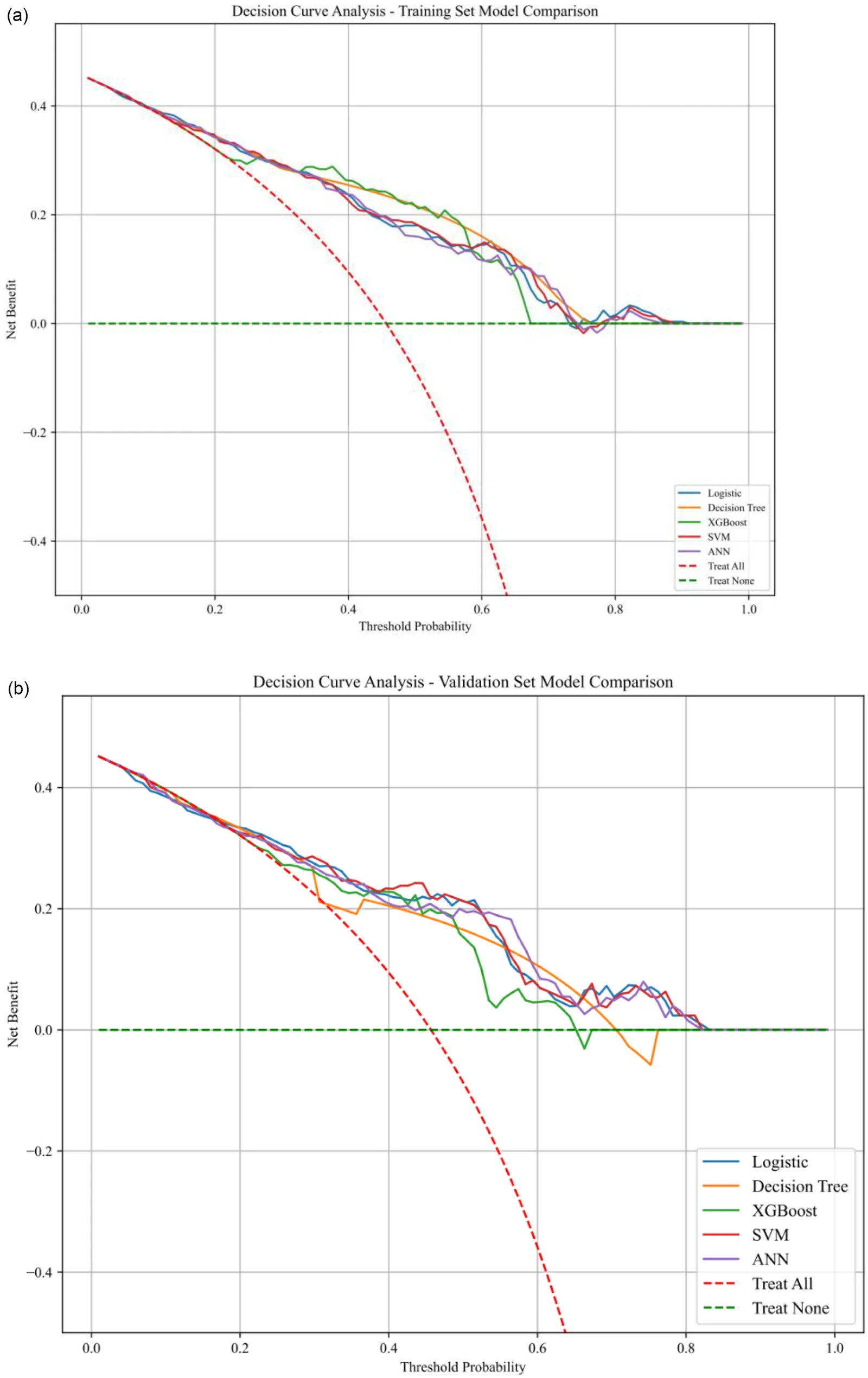
**(a)** Decision curves of five diagnostic models in the training set. **(b)** Decision curves of five diagnostic models in the validation set.

**Figure 5:**
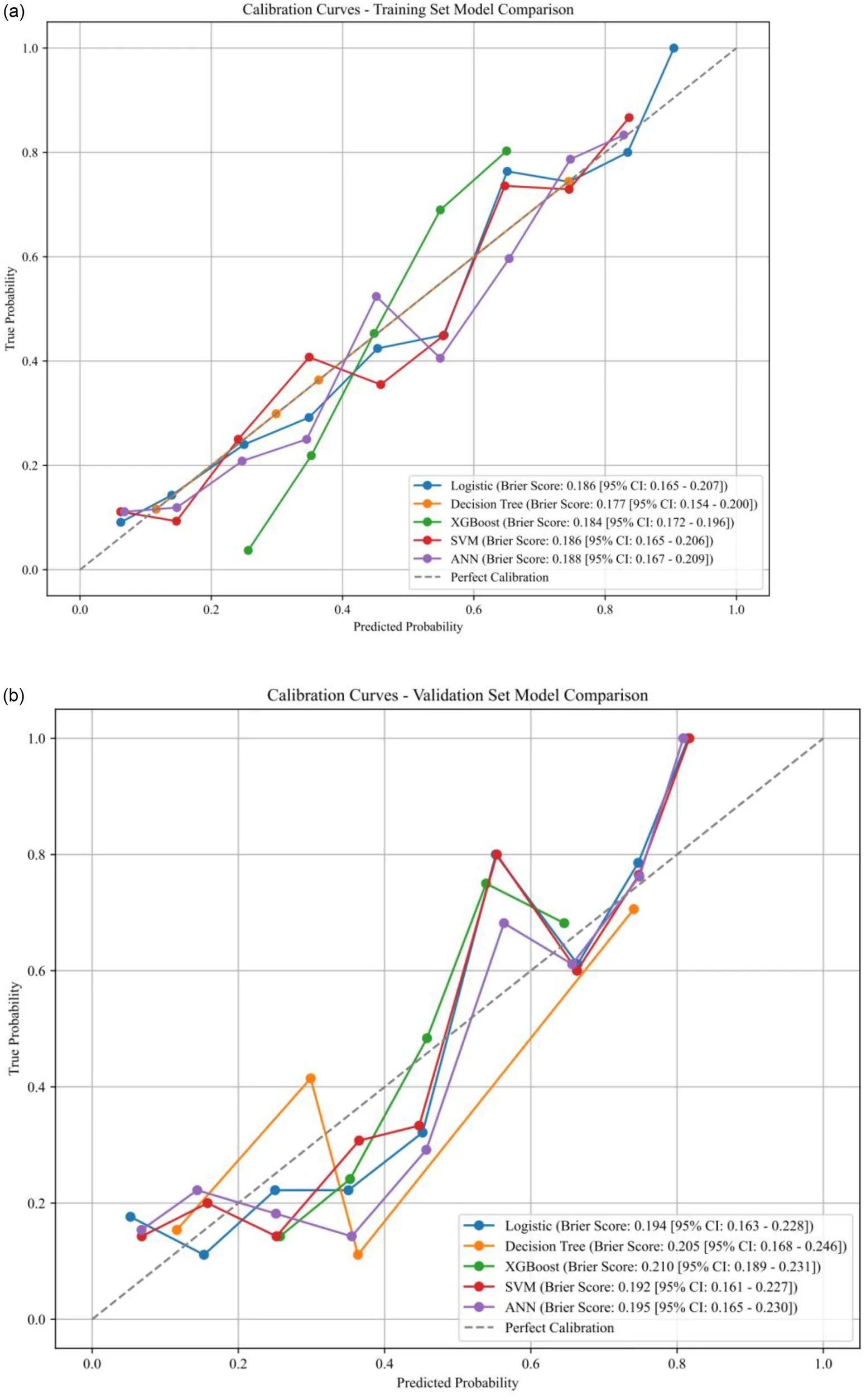
**(a)** Calibration curves of five diagnostic models in the training set. **(b)** Calibration curves of five diagnostic models in the validation set.

**Table 3:** Diagnostic performance of the five machine learning models in the training set.

Model	AUC	95% CI	Accuracy	Precision	Sensitivity	Specificity	F1 Score	Kappa	Youden's J	PPV	NPV
Logistic	0.791	[0.74, 0.841]	0.723	0.680	0.745	0.706	0.711	0.447	0.450	0.680	0.767
Decision Tree	0.791	[0.741, 0.838]	0.760	0.744	0.723	0.791	0.733	0.515	0.514	0.744	0.772
XGBoost	0.847	[0.804, 0.889]	0.760	0.752	0.708	0.804	0.729	0.514	0.512	0.752	0.766
SVM	0.791	[0.739, 0.841]	0.727	0.685	0.745	0.712	0.713	0.453	0.456	0.685	0.768
ANN	0.791	[0.738, 0.84]	0.700	0.641	0.781	0.632	0.704	0.406	0.413	0.641	0.774

**Table 4:** Diagnostic performance of the five machine learning models in the validation set.

Model	AUC	95% CI	Accuracy	Precision	Sensitivity	Specificity	F1 Score	Kappa	Youden's J	PPV	NPV
Logistic	0.785	[0.698, 0.860]	0.756	0.745	0.707	0.797	0.726	0.506	0.504	0.745	0.764
Decision Tree	0.706	[0.613, 0.791]	0.709	0.706	0.621	0.783	0.661	0.407	0.403	0.706	0.711
XGBoost	0.746	[0.656, 0.832]	0.701	0.717	0.569	0.812	0.635	0.387	0.381	0.717	0.691
SVM	0.786	[0.701, 0.862]	0.756	0.737	0.724	0.783	0.730	0.507	0.507	0.737	0.771
ANN	0.782	[0.692, 0.861]	0.740	0.698	0.759	0.725	0.727	0.480	0.483	0.698	0.781

Based on the four core variables (age, dyslipidemia, LMR, and MHR) screened out by the Boruta algorithm and VIF analysis, five machine learning models were constructed: logistic regression, decision tree, XGBoost, SVM, and ANN. In an independent validation set, the AUC of the SVM model was 0.786 (95% CI: 0.701–0.862), ranking first among all models, slightly higher than logistic regression (0.785) and ANN (0.782), and significantly higher than decision tree (0.706), and XGBoost (0.746). The DeLong test showed that, as shown in Table 5, there was no statistically significant difference in the AUC between SVM and logistic regression (*P* *=* *0.866*) and ANN (*P* *=* *0.442*), but it was significantly better than that of decision tree (*P* *=* *0.013*) and XGBoost (*P* *=* *0.007*).

**Table 5:** Pairwise comparison of AUC among models by DeLong test in the validation set.

Model 1	Model 2	AUC 1	95% CI 1	AUC 2	95% CI 2	*P* value
Logistic	Decision Tree	0.785	[0.698, 0.860]	0.706	[0.613, 0.791]	0.0121
Logistic	XGBoost	0.785	[0.698, 0.860]	0.746	[0.656, 0.832]	0.0079
Logistic	SVM	0.785	[0.698, 0.860]	0.786	[0.701, 0.862]	0.866
Logistic	ANN	0.785	[0.698, 0.860]	0.782	[0.692, 0.861]	0.6297
Decision Tree	XGBoost	0.706	[0.613, 0.791]	0.746	[0.656, 0.832]	0.1311
Decision Tree	SVM	0.706	[0.613, 0.791]	0.786	[0.701, 0.862]	0.0126
Decision Tree	ANN	0.706	[0.613, 0.791]	0.782	[0.692, 0.861]	0.0209
XGBoost	SVM	0.746	[0.656, 0.832]	0.786	[0.701, 0.862]	0.007
XGBoost	ANN	0.746	[0.656, 0.832]	0.782	[0.692, 0.861]	0.0326
SVM	ANN	0.786	[0.701, 0.862]	0.782	[0.692, 0.861]	0.4419

However, AUC only reflects the overall discriminatory ability. Comprehensive classification indicators can better demonstrate the actual diagnostic value of each model. The F1 score (0.730), Kappa coefficient (0.507), and Youden index (0.507) of SVM were all the highest among the five models, suggesting that it has advantages in the comprehensive balance of precision and recall rate, observation consistency, and sensitivity and specificity. Specifically, the sensitivity (0.724) and specificity (0.783) of SVM are relatively balanced. Although XGBoost has the highest specificity (0.812), its sensitivity is only 0.569, and the bias of missed diagnosis is obvious. Although the sensitivity of ANN reached 0.759, its specificity dropped to 0.725, increasing the risk of misdiagnosis. Both indicators of the decision tree (0.621/0.783) were not satisfactory. Although Logistic regression (0.707/0.797) was close to SVM, its F1 score (0.726) and Kappa score (0.506) were slightly inferior. In terms of probabilistic calibration, the Brier Score of SVM was 0.192 (95% CI: 0.161–0.227), the lowest among all models, and the calibration curve was closest to the ideal diagonal. Logistic regression (0.194) and ANN (0.195) followed, while the calibration deviation between XGBoost (0.210) and decision tree (0.205) was relatively large. Decision curve analysis shows that the net benefit curves of SVM, Logistic regression, and ANN almost overlap and are consistently higher than the “all treatment” and “no treatment” strategies. However, the net benefit of XGBoost fluctuates significantly within the 0.5–0.7 threshold range and drops below the zero line. When the threshold of the decision tree is above 0.7, the net benefit drops sharply, and the clinical stability is poor. In contrast, SVM remains stable across the full threshold range and provides a sustained and reliable net benefit within the commonly used clinical decision interval of 0.2 to 0.6. To sum up, although Logistic regression and ANN are similar to SVM at the AUC level, SVM shows a better balanced performance in comprehensive classification consistency, probability calibration, and clinical decision stability, while XGBoost and decision trees have obvious shortcomings in discriminative performance or clinical utility. Therefore, SVM was determined as the final recommended model.

### 3.4 Model description

The interpretability of the SVM model was evaluated based on SHAP analysis, and the results are shown in Figure 6. The global feature importance bar chart shows that Dyslipidemia makes the greatest average contribution to the model output (mean SHAP ≈ 0.12). Next are Age (mean SHAP ≈ 0.11), LMR (mean SHAP ≈ 0.05), and MHR (mean SHAP ≈ 0.03). The Beeswarm map further reveals the direction and distribution of the influence of each feature: dyslipidemia shows a typical binary effect, with SHAP values concentrated in the positive range when positive, significantly increasing the diagnostic risk of obstructive coronary heart disease (OCAD); The increase in age is consistent with the rightward shift trend of SHAP values, suggesting that advanced age is an independent risk factor. LMR shows an inverse distribution, with high LMR values corresponding to negative SHAP values, indicating a protective effect. The high values of MHR often fall in the positive range, suggesting that it promotes an increase in risk. As shown in Figure 7.

**Figure 6:**
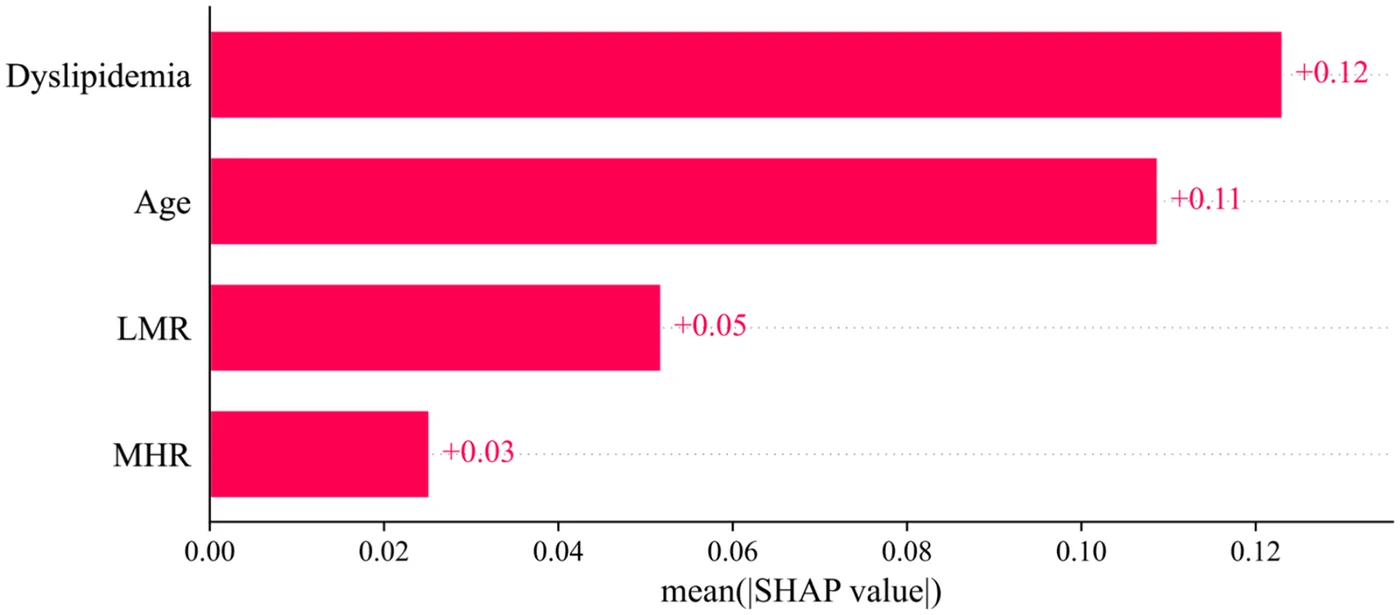
Bar plot of mean absolute SHAP values for feature global importance ranking.

**Figure 7:**
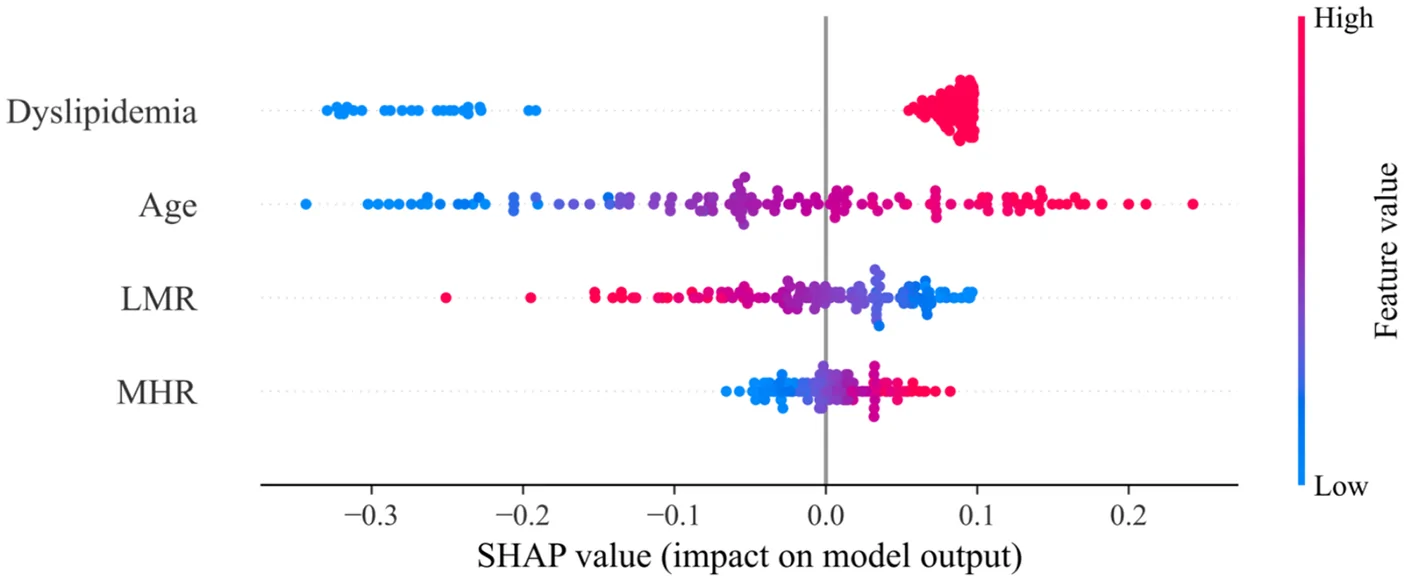
SHAP beeswarm summary plot of the five key variables.

The SHAP dependency graph quantifies the specific functional relationship between each feature and the model output. Age is approximately linearly and positively correlated with the SHAP value. The SHAP value turns from negative to positive around the age of 60, indicating that this age node is a key threshold for disease susceptibility. Beyond this age, the risk of OCAD increases independently with age. Dyslipidemia, as a binary variable, has a constant positive SHAP value when its value is 1, confirming that it is a stable risk factor. LMR was significantly negatively correlated with SHAP value. When LMR was approximately 5.0, the curve crossed the zero line. Below this threshold, the SHAP value turned positive, suggesting that a decrease in LMR would lose its protective advantage and become a risk promoter. MHR is positively correlated with the SHAP value. An increase in MHR is accompanied by a monotonically increasing SHAP value, suggesting that this lipid-inflammation composite index has a continuous risk superposition effect on OCAD. In conclusion, SHAP analysis not only verified the clinical interpretability of the SVM model, but also clarified the specific role directions and critical characteristics of age, dyslipidemia, LMR and MHR in the risk discrimination of hypertension combined with OCAD. See Figure 8.

**Figure 8:**
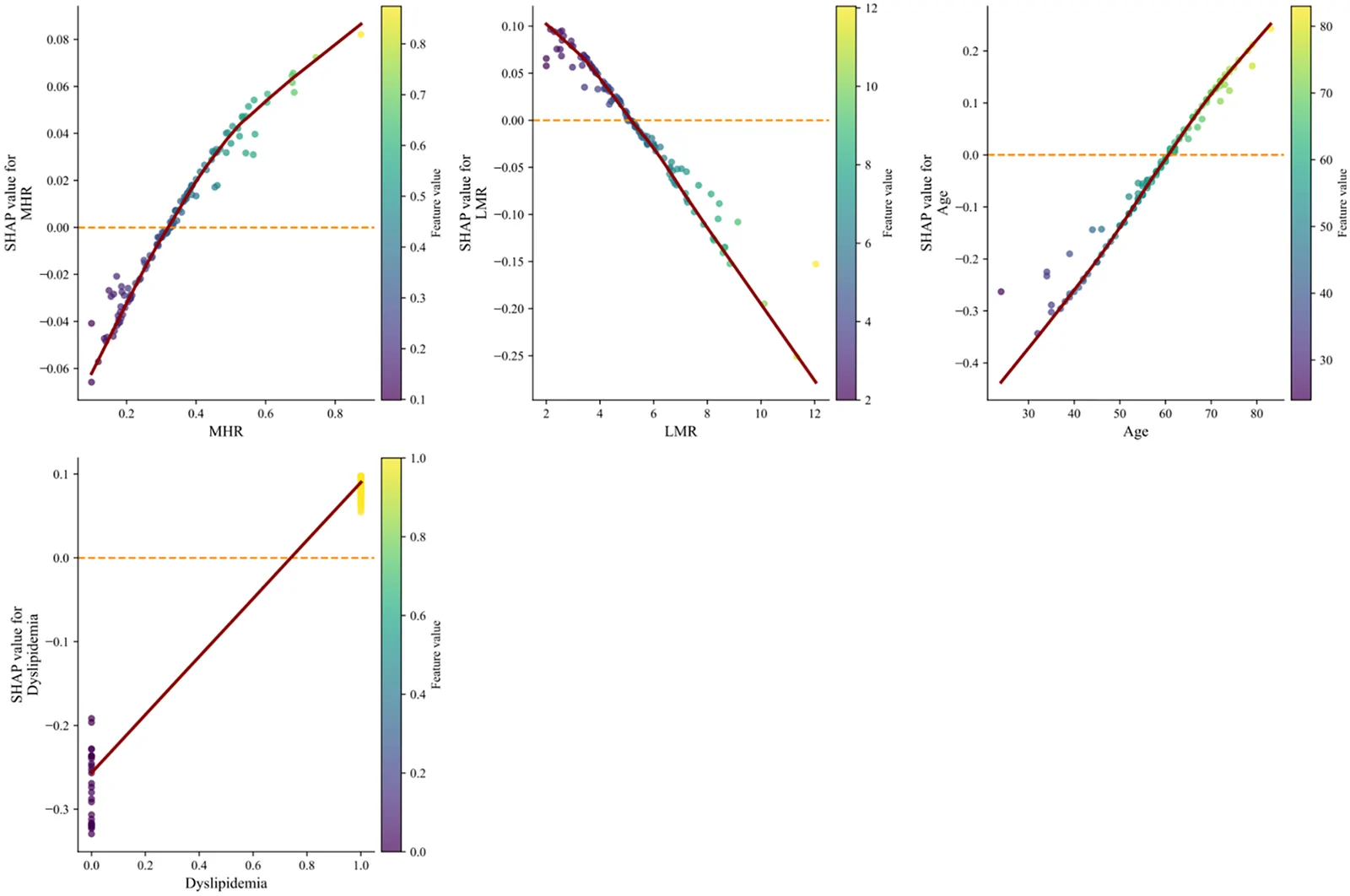
SHAP dependence plots of the four core features.

To further understand the application of the SVM model in specific patients, we conducted a diagnosis of the risk of obstructive coronary artery disease in one patient in the validation cohort. The representative case was selected as the patient whose predicted probability was closest to the median predicted probability of the validation cohort, thereby illustrating a typical risk profile. The results were visually presented through the SHAP waterfall diagram. As shown in Figure 9, the baseline expected value of this representative case is 0.539. The specific characteristics of the patient are as follows: no Dyslipidemia (Dyslipidemia = 0), 59 years old, LMR is 5.512, and MHR is 0.289. Among all the characteristics, the absence of dyslipidemia made the greatest positive contribution to the model output (SHAP = +0.29), significantly increasing the probability that this patient was classified as Class 0 (non-obstructive coronary heart disease). Age (+0.02), LMR (+0.01), and MHR (+0.01) are also positively driven, but their contribution magnitudes are relatively small. The final model output f(x) reached 0.875, suggesting that the patient has a higher probability of belonging to the non-obstructive coronary heart disease category and a lower risk of obstructive coronary heart disease.

**Figure 9:**
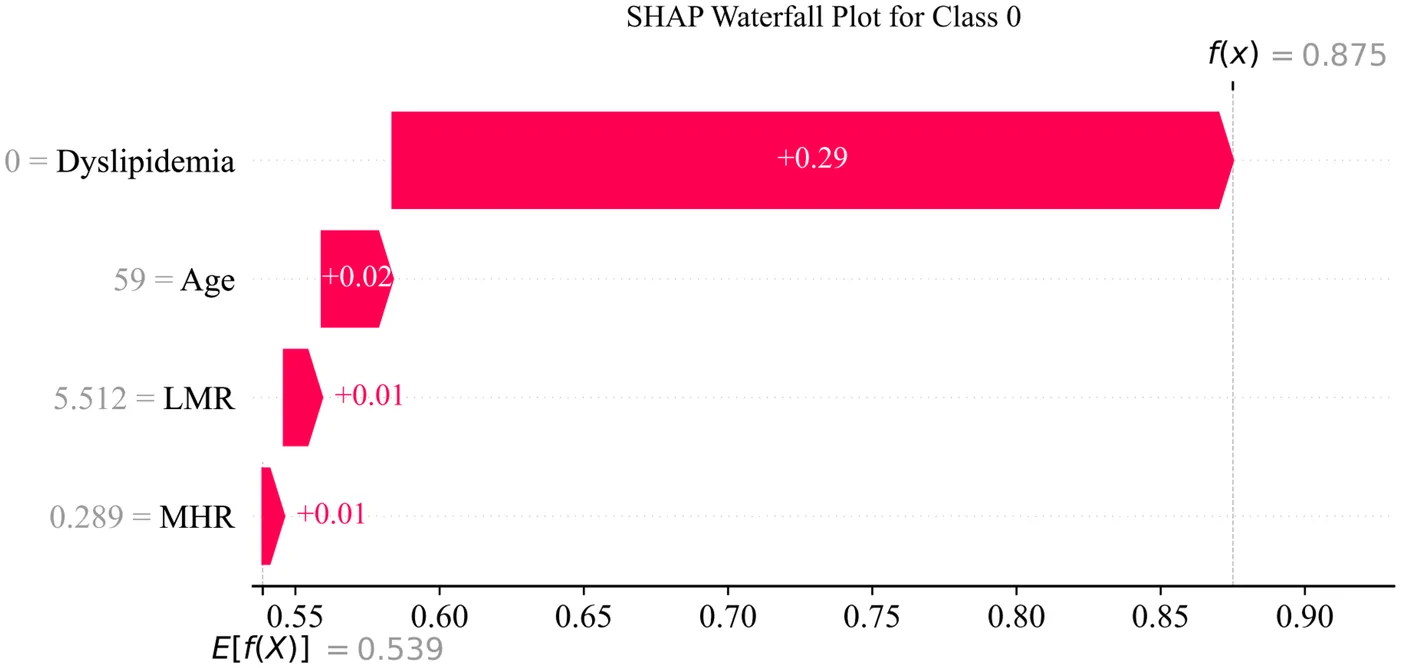
SHAP waterfall plot of a representative individual patient sample.

## 4 Discussion

In this retrospective study involving patients with hypertension, we developed and validated a machine-learning-based diagnostic model for the early identification of obstructive coronary artery disease (OCAD). Aftervariable screening using the Boruta algorithm and rigorous variance inflation factor (VIF) assessment to detect multicollinearity, an SVM model containing four core clinical predictors (age, dyslipidemia, LMR, and MHR) was constructed. The events-per-variable (EPV) value was greater than 30. In line with the standards reported by Riley et al., this sample size was sufficient to develop a stable machine learning model. The SVM model emerged as the optimal diagnostic tool, demonstrating robust generalization performance and a favorable F1 score in the validation set compared with the other models. Beyond conventional discriminative metrics, we assessed the model's calibration and clinical utility using calibration curves, decision curve analysis (DCA), and clinical impact curves. In addition, SHAP analysis was used to enhance the interpretability of the model. This study provides comprehensive evidence to support early risk stratification and informed clinical decision-making for obstructive coronary artery disease in patients with hypertension.

### 4.1 Clinical correlation and mechanism of core variables

SHAP global feature importance analysis identified a history of dyslipidemia as the two most significant predictors in the diagnostic model. Beeswarm and dependence plots further corroborated that both factors exhibited a strong positive correlation with an increased risk of obstructive coronary artery disease (CAD). This finding aligns closely with numerous studies indicating that lipid metabolic disorders contribute to the development of coronary atherosclerosis. From a mechanistic perspective, in a state of dyslipidemia, atherosclerotic lipids such as low-density lipoprotein cholesterol (LDL-C) deposit subcutaneously within blood vessels, triggering and intensifying local inflammatory responses. Meanwhile, the shear stress injury of the vascular endothelium caused by hypertension has a synergistic effect with lipid deposition, accelerating monocyte infiltration, foam cell formation, and intimal thickening, and promoting the progression of coronary artery lesions from non-obstructive to obstructive lesions with lumen stenosis of ≥50%. Therefore, dyslipidemia is not only an independent metabolic risk factor but also a key pathological bridge connecting the severity of hypertension and coronary atherosclerosis.

This study demonstrated that elevated levels of the lymphocyte-to-monocyte ratio (LMR) exert a sustained linear protective effect and are significantly negatively correlated with the likelihood of obstructive coronary artery disease (CAD). This finding aligns with prior literature indicating that LMR functions as a favorable diagnostic biomarker for coronary heart disease and hypertension. Mechanistically, atherosclerosis represents a lipid-driven inflammatory response. Monocytes serve as precursors to macrophages during the initial stages of atherosclerotic plaque formation. Experimental studies have established that monocytes and macrophages facilitate the initiation and progression of atherosclerosis through various pathways, including the activation of fibromuscular responses, the induction of endothelial injury, and the acceleration of intimal lipid accumulation. A reduced LMR directly indicates an imbalance between pro-inflammatory and anti-inflammatory responses within the body. As an inexpensive and readily accessible peripheral blood marker, LMR can serve as a sensitive indicator for the dynamic monitoring of vascular endothelial inflammation in hypertensive patients.

Elevated MHR significantly increased the diagnostic risk of obstructive coronary artery disease (CAD) among hypertensive patients in this study. Emerging evidence indicates that MHR, as a novel inflammatory–lipid composite biomarker, demonstrates superior discriminatory performance compared with single conventional parameters, such as LDL-C or HDL-C alone. Unlike isolated lipid measurements, MHR simultaneously reflects the dynamic interplay between pro-atherogenic inflammation and impaired vascular protection, offering a more integrated assessment of atherosclerotic risk (26).

From a mechanistic perspective, the absolute count of monocytes reflects the innate immune load of the body. Circulating monocytes are recruited into the subcutaneous tissue of blood vessels, differentiate into macrophages, and transform into foam cells, which is the key to the occurrence and development of atherosclerotic plaques. HDL-c reflects anti-atherosclerotic functions such as reverse transport of high-density lipoprotein cholesterol, anti-inflammation, and anti-oxidation. An increase in MHR indicates enhanced activity of inflammatory monocytes, whereas the vascular protective effect mediated by HDL-C is insufficient. Under conditions of hypertension, continuous endothelial shear stress damages vascular integrity, upregulates adhesion molecules to promote monocyte infiltration, and is often accompanied by abnormal HDL function, such as cholesterol efflovesicular and decreased activity of oxygenase. These two factors work together to accelerate lipid deposition and intimal thickening, promoting the progression of coronary artery disease from non-obstructive to obstructive. The inclusion of MHR in this study has dual clinical value. This indicator is derived from routine blood tests and lipid tests, is simple and inexpensive, and can enhance the practicality of the model without additional tests. At the same time, it can integrate inflammatory and lipid metabolism information, and more comprehensively reflect the pathological basis of atherosclerosis in hypertensive patients than a single marker. In clinical practice, monitoring MHR can screen high-risk hypertensive populations with obstructive CAD, guiding priority non-invasive coronary screening and intensive lipid regulation intervention (27, 28).

The SHAP dependence plot revealed a consistently increasing baseline risk trajectory associated with age. The SHAP value transitioned from negative to positive at approximately 60 years, indicating that advancing age independently elevated the diagnostic risk beyond this threshold. In alignment with prior research, chronic mechanical damage to the vascular endothelium resulting from hypertension exerts a significant cumulative effect over time. The incidence of obstructive lesions typically peaks after the age of 60, coinciding with a physiological decline in vascular elasticity and the accumulation of multiple cardiovascular risk factors. Persistent shear stress injury to the vascular endothelium due to hypertension compromises endothelial barrier function by downregulating the activity of endothelial nitric oxide synthase (eNOS) and upregulating the expression of adhesion molecules (29). Concurrently, aging induces elastin fragmentation and increased collagen deposition within the vascular wall, which elevates vascular stiffness and further amplifies the impact of systolic pressure on the endothelium (30). Age-related declines in antioxidant enzyme activity lead to the accumulation of reactive oxygen species (ROS) and promote the oxidative modification of low-density lipoprotein (31, 32). Dyslipidemia is characterized by reduced lipoprotein lipase activity and compromised reverse cholesterol transport (33). These mechanisms interact synergistically, with their cumulative effects reaching a clinical threshold around the age of 60. This threshold propels the progression of atherosclerosis from non-obstructive plaques (diameter stenosis < 50%) to obstructive lesions (stenosis ≥ 50%). This age period signifies a critical turning point for the advancement of coronary artery lesions in hypertensive patients.

### 4.2 Model performance and comparison with existing models

Our SVM model (AUC = 0.786) achieved moderate discrimination using only routine blood indices. Although models incorporating coronary artery calcium scores report a higher (AUC = 0.83), they require CT imaging and radiation exposure. Our model is intended for initial risk screening in resource-limited settings and not as a replacement for imaging-based diagnosis (8). Some investigations developed a single model specifically for elderly patients without comparing multiple algorithms to identify the optimal one, resulting in limited diagnostic performance. Additionally, certain studies did not achieve individualized risk assessments. Traditional regression models were often constructed using a small number of biomarkers alongside a large array of variables. Although these studies highlighted the potential of machine learning in predicting coronary artery disease among hypertensive patients (AUC = 0.906), they depended on non-routine laboratory indicators such as fibrinogen, lipoprotein(a), and prothrombin time. These parameters are typically unavailable in primary care settings or during routine physical examinations, which restricts the broader application of these models (34). In contrast, the current model is based on four routine indicators that can be readily obtained from outpatient and inpatient medical records. By employing SHAP analysis, the model elucidates the “black box” of machine learning. This approach facilitates a comprehensive assessment of feature importance at the population level and provides detailed risk interpretations for individual patients, significantly enhancing clinical acceptance and practicality.

### 4.3 Clinical significance

#### 4.3.1 Potential clinical applications of the diagnostic model

In clinical practice, the risk of obstructive coronary artery disease (CAD) in hypertensive patients is closely linked to adverse cardiovascular events, including myocardial infarction, heart failure, and cardiovascular mortality. However, the symptoms associated with this condition are often atypical and may be misattributed to concurrent issues such as blood pressure fluctuations or left ventricular hypertrophy, resulting in a delayed diagnosis. Consequently, the machine learning diagnostic model developed in this study can function as a non-invasive, cost-effective risk screening tool, aiding clinicians in the rapid identification of high-risk patients during the early outpatient or inpatient stages.

For patients identified as high-risk by the model, the diagnostic results can inform more aggressive diagnostic evaluations and intervention strategies in the future. These may include prioritizing coronary computed tomography angiography (CTA) or stress echocardiography, initiating intensive lipid-lowering and antiplatelet therapies early, and referring patients to specialized centers for coronary angiography when necessary. This approach facilitates early intervention before irreversible myocardial damage occurs in the patient. Conversely, patients classified as low-risk may avoid unnecessary invasive or costly examinations, thereby promoting rational allocation of medical resources while ensuring patient safety. Importantly, this tool is intended to support clinical decision-making and risk stratification rather than supplant physicians' professional judgment. Its outputs should be interpreted in conjunction with patients' clinical manifestations, ambulatory blood pressure monitoring, electrocardiograms, and other laboratory results.

#### 4.3.2 Potential value of the model for primary care screening and resource allocation

Unlike conventional diagnostic modalities that depend on costly, invasive, or radiation-exposing procedures such as coronary angiography and coronary CTA, our diagnostic model effectively meets the practical needs of primary care settings. It employs only four routine indicators: age, history of dyslipidemia, LMR, and MHR, enabling accurate identification of high-risk patients without the necessity for advanced imaging equipment. In community clinics, physicians can input age, dyslipidemia history, LMR, and MHR into the model. High-risk outputs (predicted probability > 0.6) trigger referral for CCTA; low-risk outputs (<0.3) support continued medical management with annual re-screening. When the model indicates a high probability of obstructive CAD, primary care physicians can promptly refer patients to facilities equipped to perform coronary CTA or angiography, thereby preventing treatment delays. Conversely, patients identified with a low predicted risk can receive optimized antihypertensive and lipid-lowering management along with regular follow-ups at primary care institutions, thus avoiding unnecessary referrals and conserving medical resources.

Moreover, the model's affordability and non-invasive characteristics support risk assessment and efficient resource distribution in managing chronic hypertension. High-risk patients may receive priority for coronary imaging, increased monitoring, and preoperative planning for revascularization. Conversely, low-risk individuals can maintain standard protocols, thereby reducing financial strain and healthcare system burdens. Importantly, the model's diagnostic likelihood reflects risk prediction using routine clinical factors, not as a replacement for gold standard pathology. Clinical practice should consider patients' overall health, guideline-recommended screening thresholds, and resource availability for informed decision-making. Additionally, the model serves as a liaison between primary care providers and specialists, offering quantitative data for tailored screening and intervention strategies.

### 4.4 Limitations

Although the model developed in this study demonstrated favorable discriminative performance, it has several limitations. First, the single-center retrospective design restricted the generalizability of our findings. All participants were recruited from a single hospital, resulting in a relatively small sample size (*n* = 427). Furthermore, the absence of multicenter or cross-regional external validation necessitates further verification of the model's geographic applicability in diverse ethnic and regional cohorts. Second, this study relied solely on cross-sectional baseline clinical data collected at admission. It did not incorporate longitudinal information, such as blood pressure variability trajectories during the progression of hypertension, dynamic fluctuations in lipid profiles, or patterns of dose adjustments for lipid-lowering agents. The integration of time-series algorithms may enhance diagnostic accuracy in future studies. Subsequent studies should incorporate these specific multimodal data to refine and improve the performance of the current diagnostic model. Third, and most importantly, this study lacks external validation in an independent cohort. The SVM model was developed and validated using a single-center dataset (*n* = 427) without external temporal or geographic validation. This limits the generalizability of our findings to other populations, regions, or healthcare settings. Although the model demonstrated satisfactory discriminative performance (AUC = 0.786) in the internal validation set, its performance in external cohorts—particularly in primary care settings with different patient demographics—remains unknown. Future multicenter prospective studies with larger sample sizes from diverse geographic and ethnic populations are urgently needed to validate and calibrate the model before any clinical implementation can be recommended. Given the absence of external validation, the current model should be considered as a proof-of-concept diagnostic tool that requires further evaluation prior to clinical deployment. Fourth, Exclusion of prior CAD patients restricts generalizability to incident disease populations. Fifth, Retrospective enrollment may introduce selection bias toward symptomatic or higher-risk patients. Sixth, A potential limitation is verification bias arising from the use of both CCTA and ICA as reference standards. While CCTA is highly sensitive for excluding obstructive CAD, it is not equivalent to ICA. The absence of a uniformly applied gold standard may have led to misclassification of a small number of true OCAD cases as NOCAD, particularly among patients with heavily calcified vessels. Future prospective studies should apply a consistent reference standard (ideally ICA) to all enrolled patients to eliminate this bias.

## 5 Conclusion

This study aimed to develop and validate a machine learning model for predicting the risk of obstructive coronary artery disease (CAD) in patients with hypertension. The results indicated that composite biomarkers, specifically LMR and MHR, in conjunction with two clinical variables—age and history of dyslipidemia—are effective for identifying obstructive CAD risk in hypertensive populations. Among the five machine learning algorithms assessed, the support vector machine (SVM) model emerged as the most robust diagnostic tool, demonstrating exceptional generalization capacity. It exhibited satisfactory discriminative power, appropriate calibration performance, and a positive clinical net benefit, with interpretability confirmed through SHAP analysis.

As a preliminary non-invasive screening tool, this model may help reduce unnecessary advanced imaging in low-risk hypertensive patients, potentially contributing to more efficient resource allocation. However, economic impact data are not available in this retrospective study, and prospective health-economic analyses are needed to validate this hypothesis.

## Data Availability

The original contributions presented in the study are included in the article/Supplementary Material, further inquiries can be directed to the corresponding author/s.

## Supplementary material

The The Supplementary Material for this article can be found online at: https://www.frontiersin.org/articles/10.3389/fcvm.2026.1922999/full#supplementary-material
